# Assessment of visceral adiposity index and lipid accumulation product index as markers of chronic kidney disease among diabetic and hypertensive patients at the Bamenda Regional Hospital, Cameroon: a cross-sectional study

**DOI:** 10.11604/pamj.2022.42.228.33499

**Published:** 2022-07-22

**Authors:** Aphrodite Tchewonpi Choumessi, Brice Ulrich Foudjo Saha, Lifoter Kenneth Navti, Ateh Sheron Tibi, Anweck Thecla Njeck, Edouard Akono Nantia

**Affiliations:** 1 Department of Biochemistry, Faculty of Science, University of Bamenda, Bambili, Cameroon

**Keywords:** Chronic kidney disease, diabetes, hypertension, lipid accumulation product index, visceral adiposity index

## Abstract

**Introduction:**

very limited studies have emphasized the importance of visceral adiposity index (VAI) and lipid accumulation product index (LAPI) in the prevention and management of chronic kidney disease (CKD) especially in diabetic and hypertension patients in developing countries including Cameroon. This study aimed at assessing whether VAI and LAPI are markers of CKD among diabetic and hypertensive patients at the Bamenda Regional Hospital, Cameroon.

**Methods:**

this analytical cross-sectional study was conducted at Bamenda Regional Hospital and involved 200 diabetic and/or hypertensive patients, including 77 males and 123 females. The participant´s anthropometric indices, biochemical parameters, VAI, LAPI, and glomerular filtration rate were investigated. A structured questionnaire was used to assess some risk factors of CKD and participant lifestyle.

**Results:**

the overweight (41%) and obesity (34%) statuses were prevalent in the population. A considerable proportion of subjects had elevated total cholesterol (46%), low-density lipoprotein (LDL) cholesterol (37.50%), triglycerides (24.5%), urea (40.5%) and creatinine (53.5%) levels. Stage 1 to 3 CKD was largely present in the elderly (>54-year-old) affecting the majority of patients (57.5%). Low education level and lack of physical activity were significantly associated with the prevalence of CKD (p < 0.001). On the contrary to creatinine (unadjusted OR = 1.36; 95% CI: 1.13-1.62), urea (unadjusted OR = 1.02; 95% CI: 1.01-1.03), HDL (unadjusted OR = 0.87; 95% CI: 0.78-0.97), total cholesterol/HDL ratio (unadjusted OR = 1.38; 95% CI; 1.12-1.71), VAI (unadjusted OR = 1.13; 95% CI: 1.05-1.22) and LAPI (unadjusted OR = 1.00; 95% CI: 1.00-1.00) were significantly associated with CKD status of the patients while HDL was negatively associated (unadjusted OR = 0.87; 95% CI: 0.78-0.97). The 9.905 and 5679 cut-offs of VAI and LAPI respectively for CKD discrimination obtained high sensitivity (75.0%) and specificity (≥79.6%).

**Conclusion:**

visceral adiposity index and LAPI were associated with CKD among diabetic and hypertensive patients. Visceral adiposity index and LAPI could be user-friendly tools for the early diagnosis of CKD among these categories of patients in Cameroon.

## Introduction

Chronic kidney disease (CKD) as defined by Kidney disease improving g outcomes (KDIGO) is considered as abnormalities of kidney structure or function, present for more than 3 months, with implications for health, based on either glomerular filtration rate lower than 60 ml/min/1.73m^2^ or markers of kidney damage, including albuminuria [1]. Chronic kidney disease is a global health issue affecting an average of 13.4% of the population worldwide, with many dying due to the high cost of treatment. The most common causes of CKD are diabetes mellitus, hypertension, and glomerulonephritis [1,2]. Chronic kidney disease is initially without symptoms and is usually detected on routine screening of blood samples by either an increase in serum creatinine or proteinuria. Later, symptoms may include leg swelling, feeling tired, vomiting, and confusion, with complications such as heart disease, high blood pressure, bone disease, and anemia [3,4]. The risk factors for this disease include a lifestyle of a high-fat, salt, protein-rich, and high-carbohydrate diet [5]. The high contribution of dietary factors emphasizes the importance of prevention in the management of CKD. Classical anthropometric index notably the body mass index (BMI) has been widely used to assess overweight/obesity because of its ease in measurement and predictive ability in younger and healthier individuals [6]. Other indices such as the waist-to-hip ratio (WHtR) and waist circumference (WC) have also been used in predicting CKD [7]. However, BMI, WHtR, and WC do not distinguish between weight from muscle and fat, between visceral and subcutaneous fat, and between peripheral and central adiposity, thereby having limitations in predicting CKD. Moreover, these obesity-related indices vary across genders, racial/ethnic, and diverse populations even across studies, and therefore remain controversial in predicting CKD among populations [6-8].

Two new obesity indices have been proposed, namely; the visceral adiposity index (VAI) and the lipid accumulation product index (LAPI). The VAI is a sex-specific measure obtained mathematically using WC, BMI, triglycerides, and high-density lipoprotein (HDL) cholesterol levels. This index has been associated with cardiovascular risk in hypertensive patients, left ventricular hypertrophy, diabetes and CKD. On the other hand, the LAPI based on a combination of WC and the fasting concentration of circulating triglycerides was equally shown to predict the incidence of diabetes, increase the risk of CVD and influence all-cause mortality [9,10]. However, little attention has been paid to the predictive efficiency of VAI and LAPI regarding CKD in diabetes and hypertensive patients in Saharan Africa. In Cameroon, the prevalence of diabetes and hypertension has been increasing from 4.7% (in 2002) and 29.6% (between 1994-2010) to 6% (in 2018) and 32.1% (between 2011-2018), respectively [11,12]. With diabetes and hypertension being among the most common causes of CKD, the increase in their prevalence in the Cameroonian population could lead to that of renal diseases. In fact, the prevalence of CKD in the country has been found to increase over time from 3 to 14% [13]. Glycemic control in known hypertensive and diabetic patients is often very poor in Cameroon and due to the asymptomatic nature of CKD, the disease is not frequently detected and individuals can lose up to 90% of their kidney function before experiencing any symptoms, resulting in loss of opportunities for prevention [14,15]. However, apart from the common epidemiological factors, no study has been carried out yet to assess the implication of VAI and LAPI as predictive markers of CKD in the Cameroonian populations. Thus, this study aimed at assessing the use of VAI and LAPI as predictive markers of CKD among diabetic and hypertensive patients attending the Bamenda Regional Hospital, North West Region of Cameroon.

## Methods

**Study design and setting:** this study was a hospital-based cross-sectional design used to ascertain and describe the variables of interest. The study was carried out at the Diabetic Unit and hemodialysis center of the Bamenda Regional Hospital from June to August 2020. It is located in Bamenda, the capital city of the North-west region of Cameroon. The hospital was given the status of a third-level reference health institution for the North-West in 2009. It serves a population now estimated at 2,180,309 inhabitants (2017 health census estimate). It has a capacity of 400 beds with a staff strength of about 440 workers.

**Inclusion and exclusion criteria:** the participants of this study were either diabetic and/or hypertensive patients in the age of majority (≥ 21 years old) attending the Bamenda Regional Hospital (BRH) who consented to take part in the study. This study only included patients who have been diagnosed and confirmed suffering from the condition for not less than 6 months. Diabetic or hypertensive patients aged ≥ 21 years suffering from chronic kidney diseases (CKD) were included in the study. A patient was considered suffering from CKD if his estimated glomerular filtrate rate (eGFR) was less than 60 mL/min per 1.73 m^2^ [16]. The patient consultation and diagnosis were done by physicians of the BRH. Patients ≥ 21 years old, and who refused to sign the consent form were not included in the study. Also, ≥ 21 years old recently diagnosed patients with less than 6 months follow-up were not included. Diabetic or hypertensive patients suffering from other diseases (HIV/AIDS, lupus, nephritis, urinary tract infection) that may exacerbate their kidney functions were excluded from this study.

**Sampling procedure:** for estimation of the sample size, we used Yamane formula [17]. This formula takes in account the total population of diabetic and hypertensive patients (N = 400) who visited the Diabetic unit of the hospital in the year 2019, and the margin of error of 0.05. This led to a minimum sample size of 200 diabetic and/or hypertensive patients. The eligible patients were conveniently sampled at the BRH.

**Data collection:** a structured questionnaire was developed and pre-tested on a small set of 15 volunteers to ensure consistency, and reliability, and to reduce intra- and inter-observer variation. The pre-tested questionnaire was administered through a face-to-face interview to the participants either in *Pidgin* English (commonly used language in the locality), English or French language, depending on the language which was best understood by the patient. The information collected in this study included demographic characteristics, lifestyle data (education and physical exercise), anthropometric parameters, lipid profile, creatinine level, urea level, blood glucose level, blood pressure, estimated glomerular filtration rate (eGFR), visceral adiposity index (VAI) and lipid accumulation product index (LAPI). Demographic characteristics consisted of age, sex, marital status, occupation, ethnicity, and education level. The anthropometric parameters included Body mass index (BMI), and waist circumference. Body mass index was obtained by calculating the weight divided by the square of the height. Weight in kilograms was measured using a digital weighing scale. The scale was calibrated to the zero level before each measurement and was tested for repeatability of the measures. Height was measured by using a stadiometer while the patient was in an upright position. Waist circumference was measured by using a flexible tape meter at both levels just above the iliac crest. Blood was collected from the antecubital vein of participants and serum obtained was used for estimation of the levels of creatinine, urea, total cholesterol, total triglycerides, and high-density lipoprotein (HDL) cholesterol according to the manufacturer (Randox laboratories Ltd, United Kingdom) instructions. In accordance with the United States National Cholesterol Education Program, adult treatment panel III (NCEP-ATP III) guidelines, abnormal lipid profile was defined as total cholesterol (TC) ≥ 200 mg/dl, HDL-cholesterol (HDL-c)< 40 mg/dl, LDL-cholesterol (LDL-c) ≥ 130 mg/dl, triglycerides ≥ 150 mg/dl, and TC/HDL-c ratio ≥ 5 [18]. Fasting blood glucose levels of participants were assessed using mylife purax glucometer (Ypsomed S.A.S, Paris, France). The current WHO diagnostic criteria for diabetes were used [19]: normal plasma glucose (fasting plasma glucose < 7.0mmol/l (126mg/dl)); diabetes (fasting plasma glucose < 7.0mmol/l (126mg/dl)). Blood pressure was measured using a mercury sphygmomanometer (adult size) by the auscultatory method. The new classification of blood pressure was used [20]: normal blood pressure (systolic blood pressure (SBP) < 120 mmHg and a diastolic blood pressure (DBP) of < 80 mmHg), elevated blood pressure (120 mmHg ≥ SBP≥ 129 mmHg and DBP < 80 mmHg), hypertension stage 1 (130 mmHg ≤ SBP ≤ 139 mmHg or 80 mmHg ≤ DBP ≤ 89 mmHg), and hypertension stage 2(SBP ≥ 140mmHg or DBP≥ 90 mmHg). The visceral adiposity (VAI) and lipid accumulation product (LAPI) indices were calculated as previously described [21,22] using the formulas below.

### Visceral adiposity index (VAI)


$$Male=\frac{WC}{39.68+(1.88\times BMI)}\times \frac{TG}{1.03}\times \frac{1.31}{HDL}$$

$$Female=\frac{WC}{36.58+(1.89\times BMI)}\times \frac{TG}{0.81}\times \frac{1.52}{HDL}$$

### Lipid accumulation product index (LAPI)


Male=(WC−65)×TG

Female=(WC−58)×TG

Where TG means triglycerides, WC= waist circumference, BMI= body mass index, HDL= high density lipoprotein. The glomerular filtrate rate (eGFR) characterizing the kidney integrity was determined according to Modification of Diet in Renal Disease (MDRD) study equation [23] using the following formulas:


$$Female=\frac{WC}{36.58+(1.89\times BMI)}\times \frac{TG}{0.81}\times \frac{1.52}{HDL}$$

$$Male:eGFR=1.86.3\times {\left(Cr\right)}^{-1.154}\times {\left(age\right)}^{-203}\times (1.210)$$

When Cr means serum creatinine.

**Data analysis:** nominal data (socio-demographic data, anthropometric data, hypertensive and diabetic status, and biochemical parameters) was expressed in terms of frequency and percent whereas continuous data (visceral adiposity index and lipid accumulation product index) was reported in terms of means and standard deviation. Bilateral unpaired t-test was used to compare CKD-negative and CKD-positive participants in terms of VAI and LAPI. Chi-square tests were performed to establish the relationship between the anthropometric parameters and CKD. Bivariate logistic regression was performed to estimate unadjusted odds ratios of factors associated with the occurrence of CKD. Receiver operating characteristics curve (ROC) analyses were used to determine the optimal cutoff values of each adiposity indices (VAI and LAPI) for CKD with the maximum Youden index (sensitivity+specificity-1). Additionally, sensitivity, specificity, negative predictive value (NPV), and positive predictive value (PPV) of these adiposity indices were used to determine their reliability as predictive markers of CKD. Statistical analysis was performed using IBM-SPSS version 25.0. Values of p<0.05 were considered as significant.

**Ethical considerations:** the study received authorization from the public health authorities (Nº: 39/ATT/NWR/RDPH), an ethical clearance (Nº: 16/APP/RDPH/RHB/IRB). Codification of data was made to assure the anonymity of the participants. Also, the design and implementation of the study followed the Helsinki Declaration for medical research.

## Results

**Socio-demographic data:** this research allowed to recruit 200 participants distributed as follows: 43 hypertensive participants (21.5%), 107 diabetic participants (53.5%), and 50 participants that were both hypertensive and diabetic (25.0%). The socio-demographic information (Table 1) of the participants showed the age group 55-64 years (31%) as the most represented. The majority of participants (70.5%) were married, and 19.5% were widows/widowers. A quarter of the participants were employed while 17% (34/200) were farmers. The majority of the participants (91%) were from the grass field area, while 2/3 had at least secondary school education.

**Table 1:** socio-demographic characteristics of enrolled participants

Socio-demographic data	N (%)
**Age** (years)	
25-34	12 (6.0)
35-44	261(3.0)
45-54	47(23.5)
55-64	62(31.0)
65-74	31(15.5)
75+	22(11.0)
**Sex**	
Female	123(61.5)
Male	77(38.5)
**Marital status**	
Single	20(10.0)
Married	141(70.5)
Widow/widower	39(19.5)
**Occupation**	
Employed	51(25.5)
Farmer	34(17.0)
House wife	14(7.0)
Other	85(42.5)
Unemployed	16(8.0)
**Race/ethnicity**	
Fulani	9(4.5)
Grass-field	182(91.0)
Other	9(4.5)
**Educational level**	
None	23(11.5)
Primary	50(25.0)
Secondary	88(44.0)
University	39(19.5)

**Anthropometric characteristics:** the anthropometric data of the participants revealed that 41% of the study population were overweight while 34% were obese with 18%, 12%, and 4% suffering from class I, II and III obesity, respectively (Table 2). More women had high (50.41%) to very high (11.38%) risk WC as compared to men (36.36 and 3.9%).

**Table 2:** anthropometric parameters of the participants

Parameters	Male	Female	Total
	N (%)	N (%)	N (%)
**Body mass index (BMI)**			
Underweight	3 (3.9)	1 (0.8)	4 (2.0)
Normal	24 (31.2)	22 (17.9)	46 (23.0)
Overweight	34 (44.2)	48 (39.0)	82 (41.0)
Class I obesity	10 (13.0)	26 (21.1)	36 (18.0)
Class II obesity	5 (6.5)	19 (15.4)	24 (12.0)
Class III obesity	1 (1.3)	7 (5.7)	8 (4.0)
**Waist circumference (WC)**			
Very low	6 (7.8)	0 (0.0)	6 (3.0)
Low	40 (51.9)	47 (38.2)	87 (43.5)
High	28 (36.4)	62 (50.4)	90 (45.0)
Very high	3 (3.9)	14 (11.4)	17 (8.5)

**Figure 1:**
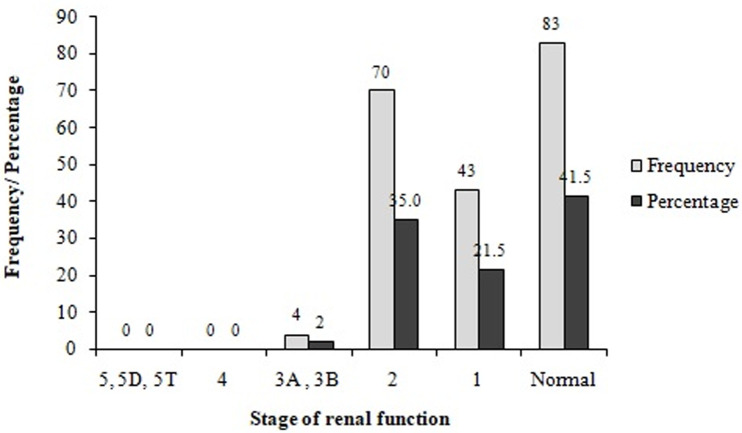
prevalence of the chronic kidney disease stages in the study population

**Figure 2:**
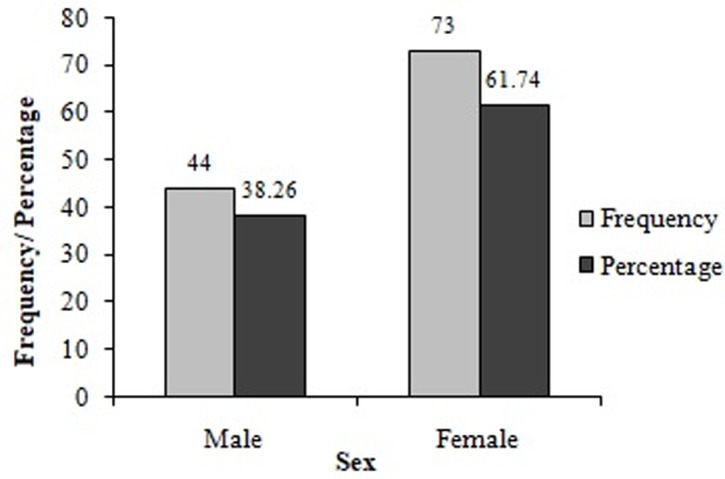
prevalence of chronic kidney disease in the study population as function of gender

**Figure 3:**
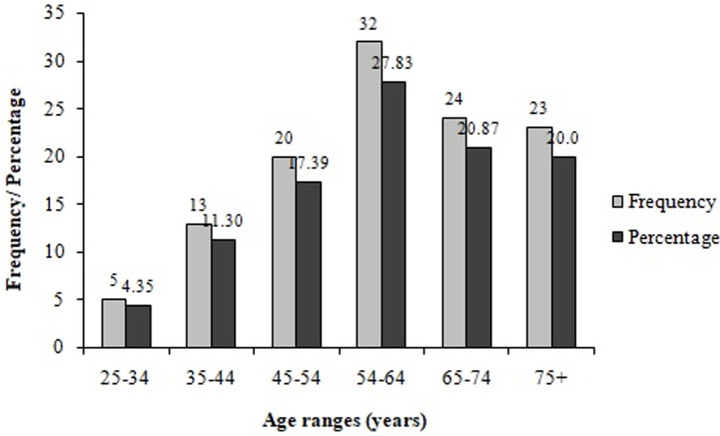
prevalence of chronic kidney disease in the study population as function of the age range

**Biochemical characteristics:** the analysis of biochemical parameters of the participants showed that almost half (46%) of participants had high total cholesterol levels (>200 mg/DL) (Table 3). Moreover, very few (8.5%, 17/200) participants had excellent total cholesterol concentrations (<125 mg/DL). Almost a quarter (23.0%) of the participants had low HDL cholesterol levels (15 – 40 mg/dL) while 37.50% (75/200) of them had high LDL cholesterol levels (131–216 mg/DL), and 24.5% experienced high triglyceride levels (150-550 mg/DL). High urea levels (> 50 mg/DL) were noticed in 40.5% of participants, while more than half (53.5%) of them had high creatinine levels (1.2–28.7 mg/DL). The participants had different stages of CKD including stages 1 (21.50%), 2 (34.0%) and 3 (2.0%), respectively (Figure 1). Out of the participants experiencing a diverse degree of CKD, 38.26% were male while the majority 61.74% were female (Figure 2). Chronic kidney disease was distributed to all age ranges with the highest prevalence found in ≥54-year-old people (Figure 3).

**Table 3:** biochemical parameters of the participants

	Frequency	Percent
**Total cholesterol**		
Excellent	17	8.5
Average	91	45.5
Very high	92	46.0
**HDL cholesterol**		
Low	46	23.0
Normal	154	77.0
**low-density lipoprotein cholesterol**		
Normal	125	62.5
Too high	75	37.5
**Triglycerides**		
Normal	151	75.5
Too high	49	24.5
**Urea (mg/dL)**		
Normal	119	59.5
Too high	81	40.5
**Creatinine (mg/dL**)		
Normal	93	46.5
Too high	107	53.5

**Figure 4:**
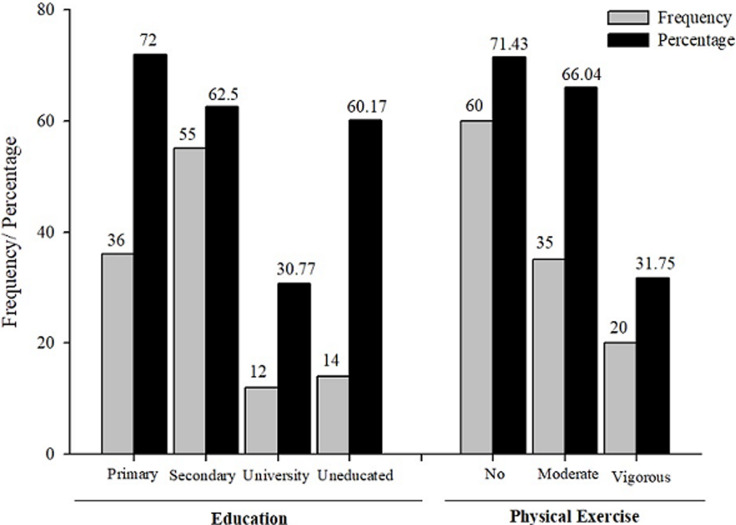
influence of education and physical activity and among chronic kidney disease participants

**Associated factors of chronic kidney diseases:** the visceral adiposity index (VAI) was significantly higher (p<0.001) in people with CKD (mean=6.48±6.86) as compared to those without CKD (mean=2.36±1.30) (Table 4). Similarly, the lipid accumulation product index (LAPI) was significantly higher (p< 0.001) in participants with CKD (mean=5488.83 ± 5252.33) when compared to those without CKD (mean=3459.59 ± 3610.46). With respect to education level, the prevalence of CKD was 60.17%, 72.0%, 61.36%, and 28.21% for illiterates, at primary, secondary and tertiary levels, respectively (Figure 4). Education significantly influenced the prevalence of CKD in the population (Chi2=18.6421, p< 0.001). Moreover, the lack of physical activity was significantly associated with the prevalence of CKD (Chi2= 11.4976, p= 0.001). The bivariate logistic regression models revealed that VAI, LAPI, creatinine, urea, HDL-cholesterol and total cholesterol/HDL ratio were found to be associated with the occurrence of CKD (Table 5). The unadjusted odds ratios (OR) of higher VAI and LAPI for CKD were respectively 1.13 (95%CI 1.05-1.22) and 1.00 (95% CI: 1.00-1.00) in all subjects. Higher levels of creatinine and urea were associated with the odds of becoming CKD positive (OR_creatinine_= 1.36; 95% CI: 1.13-1.62 and OR_urea_= 1.02; 95% CI: 1.01-1.03 respectively). A higher level of HDL-cholesterol was negatively associated with CKD occurrence (unadjusted OR = 0.87; 95% CI: 0.78-0.97) while a higher total cholesterol/HDL ratio was positively associated with CKD occurrence (OR = 1.38; 95% CI: 1.12-1.71).

**Table 4:** mean of visceral adiposity index and lipid accumulation product index of participants with and without CKD

	Visceral adiposity index (VAI)		p-value
Variable	Frequency	Mean ± SD	
CKD=No	85	2.36±1.30	p˂0.001
CKD=Yes	115	6.48±6.86	
	**Lipid accumulation product index (LAPI)**		
CKD=NO	85	3459.59 ± 3610.46	p˂0.01
CKD=Yes	115	5488.83 ± 5252.33	

**Table 5:** bivariate analysis of anthropometric and biochemical parameters affecting the chronic kidney disease occurrence

Variables	CKD	
	Unadjusted OR	p-value
Visceral adiposity index (VAI)	1.13 (1.05-1.22)	0.002
Lipid accumulation product index (LAPI)	1.00 (1.00-1.00)	0.028
Age (year)	1.35 (0.66-2.78)	0.409
**Sex**		
Female	1.61 (0.22-11.70)	0.636
Male	1	
**Waist circumference (WC)**		
Abnormal	1.15 (0.12-11.35)	0.904
Normal	1	
**Body mass index (BMI)**		
Abnormal*	3.17 (0.44 –23.13)	0.255
Normal	1	
Creatinine (mg/dl)	1.36 (1.13-1.62)	0.001
Urea (mg/dl)	1.02 (1.01-1.03)	0.001
Total cholesterol (mg/dl)	1.01 (0.99-1.02)	0.291
High-density lipoprotein-cholesterol (mg/dl)	0.87 (0.78-0.97)	0.013
low-density lipoprotein-cholesterol (mg/dl)	1.00 (0.99-1.01)	0.537
Triglycerides (mg/dl)	1.00 (0.99-1.01)	0.859
Total cholesterol/HDL ratio	1.38 (1.12-1.71)	0.003

* abnormal in this case considers both under nutrition (BMI < 18.5) and over nutrition (BMI>24.9)

**Figure 5:**
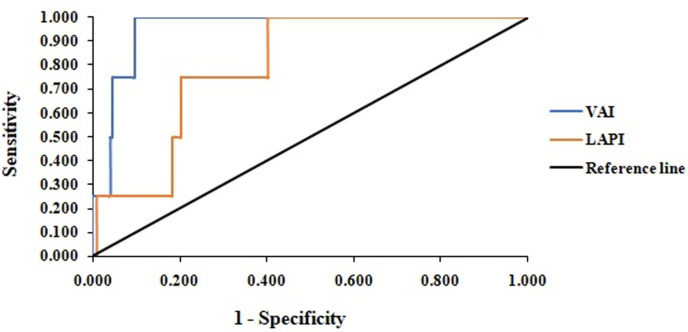
receiver operating characteristics curve (roC) of visceral adiposity index (VAI) and Lipid accumulation product index (LAPI) as predictors of chronic kidney diseases

**Sensitivity and specificity of VAI and LAPI:** for both curves and using Youden index, the best cut-off points for VAI and LAPI in the identification of chronic kidney diseases (CKD) were 9.905 and 5679, respectively (Figure 5). For CKD classification criteria, the area under the receiver operating characteristic curve for both VAI and LAPI was equal or greater than 0.8 (Table 6). The proportion of participants with CK Visceral adiposity index was more able (specificity) to correctly identify participants without CKD that LAPI. The probability that participants with VAI ≥ 9.905 or LAPI ≥ 5679 developed CKD was very unlikely (PPV 25.0% and 7.0%, respectively), and even after a negative test, the disease could not be ruled out with a residual probability of 0.5% and 0.6% (NPV 99.5% and 99.6%) for VAI and LAPI, respectively.

**Table 6:** sensitivity, specificity, negative predictive value (NPV), positive predictive value (PPV), and area under the ROC (AUROC) of the 9.905 cut off for visceral adiposity index (VAI) and the 5679 cut off for lipid accumulation product index (LAPI) as indicators of chronic kidney disease

Indicator	AUROC	Sensitivity	Specificity	NPV	PPV
VAI	0.954	75.0	95.4	99.5	25.0
LAPI	0.800	75.0	79.6	99.4	7.0

## Discussion

Chronic kidney disease (CKD) is a chronic renal disease that has been related to health dysfunctions including diabetes mellitus, hypertension, and glomerulonephritis [1,2]. Though CKD is currently on the rise worldwide, including in developing countries, prediction or prevention remains a key aspect of curving down this disease [24]. The present study involved diabetic and hypertensive patients with the goal of assessing visceral VAI and LAPI as predictive markers of CKD. Some factors such as alcohol and cigarette consumption were noticeable in the study population. Consistently, studies have shown significant influence of these factors on the incidence of cardiovascular diseases including diabetes and hypertension [25,26]. Anthropometric parameters showed that the majority of the population was made up of either overweight or obese patients. The general high risk (high and very high) waist circumference was prevalent in females. Similarly, literature reports commonly support overweight and obesity as determinants of cardiovascular diseases [27,28]. The extent of these anthropometric parameters was consistent with the considerable percentage of subjects with high total cholesterol, high LDL cholesterol, and triglycerides levels. Moreover, almost half of the participants showed high urea and creatinine levels, which is consistent with a study on hypertensive patients treated at Wahidin Sudirohusodo Hospital, Makassar in Indonesia [29,30]. Stage 1 to 3 CKD was present in the majority of the patients, and this health issue was more present among those above 54 years of age. This finding is consistent with a previous report, which indicated that there is a decrease in renal function with aging [31]. In fact, renal mass decreases with 30% of the glomeruli destroyed with aging, with the steepest decline observed from the age of 50. Also, age is associated with some risk factors (obesity, hypertension, and diabetes mellitus) of CKD. Consequently, creatinine clearance declined at a higher rate among older individuals with regard to younger people, and this results in a higher prevalence of CKD in older participants [32,33].

Contrary to anthropometric parameters (BMI, WC, WHtR), VAI and LAPI were significantly associated with CKD. VAI and LAPI had the ability of identifying hypertensive and (or) diabetic patients with CKD, as these indices were higher in people with CKD as compared to those without CKD. These findings are in agreement with a study carried out among the rural population in Northeast China on VAI and LAPI as indices to identify CKD [34]. This association between VAI and LAPI with CKD could be explained by the fact that visceral obesity is closely associated with metabolism [35]. Visceral adipose tissue when combined with anthropometric measurements (such as WC and BMI) and blood biochemical indexes such as serum triglyceride levels and high-density lipoprotein cholesterol levels, exhibits consistency in the evaluation of CKD [17]. Metabolically, CKD dysregulates normal triglycerides and cholesterol metabolism, as such increases triglyceride levels and low HDL cholesterol levels with only mild increase, normal or low levels of LDL cholesterol, all which were noticed in our study population. The dysregulation in triglycerides is due to a decrease in their hepatic triglyceride lipoprotein lipases, as well as a decrease in the number of receptors and an alteration in the lipoprotein substrates in the patients [36]. Higher sensitivity of both VAI and LAPI was associated with higher negative predictive value. This confirmed the corollary that higher sensitivity goes with higher negative predictive value, in other words, the test performs better as a “rule-out” test [37]. This means that at higher VAI and LAPI cutoffs, more subjects who were actually CKD-positive had a positive test result on a high sensitivity test and there were fewer false negatives. Conversely, lower VAI and LAPI cutoffs were highly specific in their ability to designate a subject who does not have a disease as negative. However, lower VAI and LAPI cutoffs exhibited poor “rule-in” test (PPV≤25.0%) indicating that many of the positive results from these testing procedures were false positives. Thus, it will be necessary to follow up any positive result with a more reliable test to obtain a more accurate assessment whether CKD is present. On average, the VAI and LAPI were higher in women than in men, though this difference was not significant. This finding is not in line with a previous study on the southern Chinese population that found a strong correlation between VAI scores and CKD in females but not in males [38]. Also, Palmer *et al*. [39] observed more abdominal obesity in women than men. The difference with the present findings could be explained by the fact that all participants in this study were registered at the diabetic clinic, routine checks and close follow up were done, and medications were given to control cholesterol levels for those who showed high values which could have possibly affected values of VAI. The mechanisms underlying the gender-specific differences of VAI and LAPI could be explained by the fact that different sex hormones might act on fat distribution which subsequently affects the association between obesity and CKD. Female hormones mainly estrogens can regulate adipose deposition and function in females affecting fat deposition and waist circumferences [40].

In the present study, factors such as education and physical activity were inversely statistically associated with the prevalence of CKD in the study population. These findings are consistent with the previous report on the association of unhealthy lifestyle behaviors with CKD and the lack of habitual moderate exercise, and daily physical inactivity among men receiving their periodic health check at a health care center in Fukuoka University in Japan [41]. In fact, education gives individuals knowledge that enabled them to improve on personal health choices and equally improved earning power, and better access to quality health care. Education equally helped individuals access information and recognize the health implications of behaviors such as alcohol consumption and cigarette smoking, helping the individuals of this category to take actions that optimize their health and that of their children, and to properly manage their illnesses [42]. Similarly, lack of habitual exercise and decreased physical activity influence the development of CKD through obesity due to the accumulation of low-density lipoprotein cholesterol, promotion of hypertension, and type 2 diabetes [43]. The present study was a cross-sectional design study covering the participant´s variables within a given punctual period of time. However, many factors in patient life such as medications, food intake as well as socio-environmental factors are susceptible to influence the occurrence and progression of CKD. Therefore, the temporal relation between risk factors and CKD is limited and only a longitudinal design study could provide better indications to disease management. Also in the present study, VAI and LAPI as indicators were unable to distinguish between positive and false positive results. Moreover, due the outbreak of the Coronavirus pandemic and increasing insecurity in the North West region of the country, many participants across the country could not attend the hospital and consequently, the present findings are not representative of the patients suffering from CKD across the country.

## Conclusion

Findings from this study supported VAI and LAPI as good markers in identifying CKD among diabetic and hypertensive patients, despite the low control on false positives (poor “rule-in” tests). Educational level and physical exercise were positively associated with CKD occurrence among diabetic and hypertensive patients. Medical practices should be encouraged to incorporate VAI and LAPI as early detection tools of CKD among diabetic and hypertensive patients.

### What is known about this topic

- Diabetes and hypertension are common risk factors of chronic kidney disease (CKD);
- Traditional anthropometric indices have shown some limitations in predicting the occurrence of chronic kidney disease;
- Visceral adiposity index (VAI) and lipid accumulation product index (LAPI) are new indices that have been proposed to better prevent CKD burden among diabetic and hypertensive patients.

### What this study adds

- Visceral adiposity index and lipid accumulation product index were good markers in identifying chronic kidney disease among diabetic and hypertensive Cameroon patients;
- These indices shall be incorporated to commonly used parameters for better management of chronic kidney disease in the local settings.

## References

[1] Levey SA, Eckardt K-U, Tsukamoto Y, Levin A, Coresh J, Rossert J (2005). Definition and classification of chronic kidney disease: a position statement from Kidney disease: improving global outcomes (KDIGO). Kidney Int.

[2] Global burden of disease (2015). Disease and injury incidence and prevalence, collaborators, global regional, and national incidence, prevalence, and years lived with disability for 310 diseases and injuries. Lancet.

[3] Liao MT, Sung CC, Hung KC, Wu CC, Lo L, Lu KC (2012). Insulin resistance in patients with chronic kidney disease. J Biomed Biotechnol.

[4] Chen TK, Knicely DH, Grams ME (2019). Chronic kidney disease diagnosis and management: a review. JAMA.

[5] Palmer SC, Maggo JK, Campbell KL, Craig JC, Johnson DW, Sutanto B (2017). Dietary interventions for adults with chronic kidney disease. Cochrane Database Syst Rev.

[6] World Health Expert Consultation (2004). Appropriate body-mass index for Asian populations and its implications for policy and intervention strategies. Lancet.

[7] Elsayed FE, Sarnak JM, Tighiouart H, Griffith LJ, Kurth T, Salem ND (2008). Waist to hip ratio, body mass index and subsequent kidney disease and death. Am J Kidney Dis.

[8] Liu J, Chen Z, Li W, Xu G, Liu J, Yi B (2016). Obesity indices for prediction of chronic kidney disease: a cross-sectional study in 26,655 Chinese adults. Zhong Nan Da Xue Xue Bao Yi Xue Ban.

[9] Bozorgmanesh M, Hadaegh F, Azizi F (2010). Diabetes prediction, lipid accumulation product, and adiposity measures; 6-year follow-up: tehran lipid and glucose study. Lipids Health Dis.

[10] Xu X, Zhao Y, Zhao Z, Zhu S, Liu X, Zhou C (2016). Correlation of visceral adiposity index with chronic kidney disease in the people's Republic of China: to rediscover the new clinical potential of an old indicator for visceral obesity. Ther Clin Risk Manag.

[11] Bigna JJ, Nansseu JR, Katte JC, Noubiap JJ (2018). Prevalence of prediabetes and diabetes mellitus among adults residing in Cameroon: a systematic review and meta-analysis. Diabetes Res Clin Pract.

[12] Defo KB, Mbanya JC, Kingue S, Tardif J-C, Choukem SP, Perreault S (2019). Blood pressure and burden of hypertension in Cameroon, a microcosm of Africa: a systematic review and meta-analysis of population-based studies. J Hypertens.

[13] Aseneh BJ, Kemah AB-L, Mabouna S, Njang EM, Ekane MSD, Agbor NV (2020). Chronic kidney disease in Cameroon: a scoping review. BMC Nephrol.

[14] Locatelli F, Vecchio LD, Pozzoni P (2002). The importance of early detection of chronic kidney disease. Nephrol Dial Transplant.

[15] Assah FK, Mbanya JC (2009). Diabetes in sub-Saharan Africa-overview of a looming health challenge. Eur Endocrinol.

[16] Ma YC, Zuo L, Chen JH, Luo Q, Yu XQ, Li Y (2006). Modified glomerular filtration rate estimating equation for Chinese patients with chronic kidney disease. J Am Soc Nephrol.

[17] Yamane T (1967). Statistics, an introductory analysis, 2^nd^ Ed.

[18] Expert Panel on Detection Evaluation (2001). Executive summary of the third report of the national cholesteroleducation program (NCEP) expert panel on detection, evaluation, andtreatment of high blood cholesterol in adults (adult treatment panel III). JAMA.

[19] Unwin N (2006). Definition and diagnosis of diabetes mellitus and intermediate hyperglycemia: report of a WHO/IDF consultation.

[20] Whelton PK, Carey RM, Aronow WS, Donald E, CaseyJr Karen JC (2018). 2017 ACC/AHA/AAPA/ABC/ACPM/AGS/APhA/ASH/ASPC/NMA/PCNA guideline for the prevention, detection, evaluation, and management of high blood pressure in adults: a report ofthe American College of Cardiology/American Heart Association task force on clinical practice guidelines. J Am Coll Cardiol.

[21] Amato MC, Giordano C, Galia M, Criscimanna A, Vitabile S, Midiri M (2010). Visceral adiposity index: a reliable indicator of visceral fat function associated with cardiometabolic risk. Diabetes Care.

[22] Mirmiran P, Bahadoran Z, Azizi F (2014). Lipid accumulation product is associated with insulin resistance, lipid peroxidation, and systemic inflammation in type 2 diabetic patients. Endocrinol Metab (Seoul).

[23] Salgado JV, Neves FA, Bastos MG, França AK, Brito DJ, Santos EM (2010). Monitoring renal function: measured and estimated glomerular filtration rates-a review. Braz J Med Biol Res.

[24] Nugent RA, Fathima SF, Feigl AB, Chyung D The burden of chronic kidney disease on developing nations: a 21^st^.

[25] Mukamal JK (2006). The effects of smoking and drinking on cardiovascular disease and risk factors. Alcohol Res Health.

[26] Shi L, Shu X-O, Li H, Cai H, Liu Q, Zheng W (2013). Physical activity, smoking, and alcohol consumption in association with incidence of type 2 diabetes among middle-aged and elderly Chinese men. PLoS One.

[27] Wilson PW, D'Agostino RB, Sullivan L, Parise H, Kannel WB (2002). Overweight and obesity as determinants of cardiovascular risk: the Framingham experience. Arch Intern Med.

[28] Channanath AM, Farran B, Behbehani K, Thanaraj AT (2015). Association between body mass index and onset of hypertension in men and women with and without diabetes: a cross-sectional study using national health data from the State of Kuwait in the Arabian Peninsula. BMJ Open.

[29] Bamanikar AS, Bamanikar AA, Arora A (2016). Study of serum urea and creatinine in diabetic and nondiabetic patients in a tertiary teaching hospital. J Med Res.

[30] Hutapea DR, Widaningsih Y, Mangarengi F, Muhadi D (2021). Analysis of urea, creatinine, and platelet indices in hypertensive patients. Indones J Clinical Pathol Med Laboratory.

[31] Nitta K, Okada K, Yanai M, Takahashi S (2009). Aging and chronic kidney disease. Semin Nephrol.

[32] Prakash S, O´Hare AM (2009). Interaction of aging and chronic kidney disease. Semin Nephrol.

[33] Kazancioğlu R (2013). Risk factors for chronic kidney disease: an update. Kidney Int Suppl 2011.

[34] Dongxue D, Yee C, Yintao C, Shuang C, Shasha Y, Xiaofan G (2016). Visceral adiposity index and lipid accumulation product index; two alternate body indices to identify chronic kidney disease among the rural population in Northeast China. Int J Environ Res Public Health.

[35] Huffman MD, Barzilai N (2009). Role of visceral adipose tissue in aging. Biochim Biophys Acta.

[36] Vaziri ND (2006). Dyslipidemia of chronic renal failure: the nature, mechanisms, and potential consequences. Am J Physiol Renal Physiol.

[37] Huang J, Zhou C, Li Y, Zhu S, Liu A, Shao X (2015). Visceral adiposity index, hyper triglyceridemic waist phenotype and chronic kidney disease in a southern Chinese population: a cross-sectional study. Int Urol Nephrol.

[38] Tripathy JP, Thakur JS, Jeet G, Chawla S, Jain S, Prasad R (2016). Urban rural differences in diet, physical activity and obesity in India: Are we witnessing the great Indian equalisation? results from a cross-sectional STEPS survey. BMC Public Health.

[39] Palmer BF, Clegg DJ (2015). The sexual dimorphism of obesity. Mol Cell Endocrinol.

[40] Florkowski CM (2008). Sensitivity, specificity, receiver-operating characteristic (ROC) curves and likelihood ratios: communicating the performance of diagnostic tests. Clin Biochem Rev.

[41] Michishita R, Matsuda T, Kawakami S, Kiyonaga A, Tanaka H, Morito N (2016). The association between unhealthy lifestyle behaviors and the prevalence of chronic kidney disease in middle age and older men. J Epidemiol.

[42] Hahn AR, Truman IB (2015). Education improves public health and promotes health equity. Int J Health Serv.

[43] Heiwe S, Jacobson SH (2014). Exercise training in adults with CKD: a systematic review and meta-analysis. Am J Kidney Dis.

